# One-Year Real-World Study on Comparison among Different Continuous Subcutaneous Insulin Infusion Devices for the Management of Pediatric Patients with Type 1 Diabetes: The Supremacy of Hybrid Closed-Loop Systems

**DOI:** 10.3390/ijerph191610293

**Published:** 2022-08-18

**Authors:** Bruno Bombaci, Stefano Passanisi, Angela Alibrandi, Giulia D’Arrigo, Serena Patroniti, Simona Averna, Giuseppina Salzano, Fortunato Lombardo

**Affiliations:** 1 Department of Human Pathology in Adult and Developmental Age “Gaetano Barresi”, University of Messina, Via Consolare Valeria 1, 98124 Messina, Italy; 2 Unit of Statistical and Mathematical Sciences, Department of Economics, University of Messina, 98124 Messina, Italy

**Keywords:** continuous glucose monitoring, glycemic variability, insulin pumps, time in range, therapy

## Abstract

Since their advent in daily clinical practice, continuous subcutaneous insulin infusion (CSII) systems have been increasingly improved, leading to a high percentage of both adult and pediatric patients with diabetes now using insulin pumps. Different types of CSII systems are currently available, which are characterized by different settings and technical features. This longitudinal observational study aims to evaluate real-word glycemic outcomes in children and adolescents with type 1 diabetes using three different CSII devices: hybrid closed-loop (HCL) systems, predictive low glucose (PLGS) systems, and non-automated insulin pumps. The secondary objective was to identify clinical variables that may significantly influence the achievement of therapeutic goals in our study cohort. One-hundred-and-one patients on CSII therapy attending our pediatric diabetes center were enrolled. When compared with the non-automated group, patients using HCL systems showed higher levels of time in target glucose range (*p* = 0.003) and lower glucose variability (*p* = 0.008). Similarly, we found significantly better glucose metrics in HCL users in comparison to PLGS patients (time in range *p* = 0.008; coefficient of variation *p* = 0.009; time above 250 mg/dL *p* = 0.007). Multiple linear regression models showed that HCL systems (time in range *p* < 0.001) and high daily percentage of glycemic sensor use (time in range *p* = 0.031) are predictors for good glycemic control. The introduction and increasing availability of novel technologies for diabetes represent a promising strategy to improve glycemic control and quality of life in pediatric patients with type 1 diabetes. Our real-world data confirm the superiority of HCL systems in terms of improvement of time spent in the target glucose range, prevention of hypoglycemia, and reduction of glycemic variability.

## 1. Introduction

Type 1 diabetes (T1D) is a chronic disease characterized by deficiency of insulin secretion [1]. According to the latest epidemiological data, over 100,000 people under 15 years are diagnosed with T1D each year [2]. Management of patients with T1D consists of intensive insulin therapy associated to a balanced diet and regular physical activity. Intensive insulin therapy is based on two different treatment types, which are multiple daily injections (MDI) and continuous subcutaneous insulin infusion (CSII). The latter, through the modulation of the insulin basal rate, allows to simulate physiological meal-independent insulin secretion and its daily variations. Moreover, a bolus can be delivered before meals or in the event of hyperglycemia [3]. Two types of CSII systems are currently available: those characterized by the presence of an infusion set that is connected to a pump through a catheter, and the so-called “patch pumps” that are directly fixed to the skin at the insertion site [4]. Benefits related to the use of CSII therapy include the achievement of lower levels of glycated hemoglobin (HbA1c), the lowering of blood glucose variability, and the reduction of hypoglycemic events [5]. Improvement in quality of life has also been demonstrated for patients using insulin pumps and their families [6]. An appropriate knowledge and correct instruction is fundamental for patients and their caregivers, both actively involved in the management of these devices. In the last two decades, the reliability of pump technology has greatly improved, leading to an increasing percentage of patients with diabetes on CSII therapy [7]. In the meantime, continuous glucose monitoring (CGM) systems have been developed and have become the standards of monitoring and care for people suffering from diabetes [8]. The introduction of sensor-augmented pump (SAP) therapy allowed to integrate glucose monitoring and insulin therapy through the use of decision-making algorithms that adjust insulin delivery in relation to sensor glycemic levels in real-time [9]. SAPs can be distinguished in low glucose suspend (LGS) systems, predictive low glucose suspend (PLGS) systems, and hybrid closed-loop (HCL) systems [10]. LGS systems stop insulin infusion when sensor glucose levels drop beyond an established hypoglycemic threshold [11,12]. PLGS systems use algorithms that can predict the occurrence of hypoglycemia and pre-emptively suspend insulin delivery [10]. Finally, the most innovative HCL systems automatically adjust insulin doses on the basis of CGM data [13,14,15].

The aim of the present study was to investigate real-word glycemic outcomes in pediatric patients with T1D using different CSII devices. The secondary objective was to evaluate clinical variables that may significantly influence the achievement of therapeutic goals in our study cohort.

## 2. Materials and Methods

In this longitudinal observational study, we recruited children and adolescents with T1D on CSII therapy attending the pediatric diabetes outpatient service of our tertiary-care center from January to March 2021. The study was realized in compliance with the Helsinki Declaration, good clinical practice, and all applicable laws and regulations. The study was not subject to ethical committee approval since it was limited to anonymized and unidentifiable data routinely collected at our diabetes center. Inclusion criteria were: diagnosis of T1D made according to the latest International Society for Pediatric and Adolescent Diabetes Clinical Practice Consensus Guidelines [16], CSII therapy for at least 6 months before the enrollment, and the use of real time or intermittently scanned CGM systems. Exclusion criteria were: partial clinical remission in accordance with the Hvidovre Study Group definition [17], occurrence of skin disorders compromising the use of insulin pumps, use of steroids or other drugs known to have a relevant impact on glycemic control, and the presence of psychological or environmental factors interfering with the proper use of CSII systems. All patients using CSII devices from our center received, along with their caregivers, an extensive training that consists of at least two visits with medical and technical staff, followed by strict remote monitoring of CGM data from the center for the first period of use. Recruited patients were followed during the entire study period, which lasted for 1 year. 

At the enrollment appointment, the following demographic and clinical data of study participants were collected: age, gender, duration of disease, auxological parameters, duration of CSII therapy, and type of insulin pumps. Three different CSII systems were considered: non-automated insulin pump (i.e., Omnipod DASH^®^, Accu-Chek™ Solo), PLGS (i.e., Medtronic MiniMed™ 640 G, Tandem t:slim X2™ Basal IQ), and HCL (i.e., Medtronic MiniMed™ 670 G, Medtronic MiniMed™ 780 G, Tandem t:slim X2™ Control IQ). Glycemic indicators were evaluated at 3, 6, 9, and 12 months after enrollment. HbA1c levels were measured at each follow-up appointment on a DCA Vantage Analyzer (Siemens^®^, Tarrytown, NY, USA). Data from continuous and flash glucose monitoring systems related to the last 90 days before the appointment were extracted from specific web-cloud platforms (i.e., Carelink software, Libreview platform, Dexcom Clarity, Diasend platform). The following glucose metrics were gathered: mean and standard deviation score (SDS) of blood glucose levels, time expressed in percentage in the ideal range of glucose between 70 and 180 mg/dL (%TIR), time expressed in percentage above 180 mg/dL (%TAR > 180 mg/dL), time expressed in percentage between 180 and 250 mg/dL (%TAR 180–250), time expressed in percentage above 250 mg/dL (%TAR > 250 mg/dL), time expressed in percentage below 70 mg/dL (%TBR < 70 mg/dL), time expressed in percentage between 54 and 70 mg/dL (%TBR 54–70 mg/dL), time expressed in percentage below 54 mg/dL (%TBR < 54 mg/dL), glucose management indicator (GMI), coefficient of variation (CV) expressed in percentage, and daily sensor use expressed in percentage. Data on insulin therapy including total daily dose per day and its distribution between basal and bolus amount were also collected. Finally, data acquired at each quarterly follow-up appointment were aggregated to obtain indicators of glycemic control relating to the whole period of study.

### Statistical Analysis

Numerical data were expressed as mean and SD, and categorical variables as absolute frequencies and percentages. The parametric approach was used since the Kolmogorov–Smirnov test demonstrated that the numerical variables were normally distributed.

In order to identify possible significant differences, with reference to all examined parameters, the one-way ANOVA test was applied between the three different types of insulin pumps (non-automated, PLGS, and HCL); post-hoc two-by-two comparisons between groups were performed using the Scheffè test. For this analysis, Bonferroni’s correction was applied and the “adjusted” alpha level was obtained by dividing the significance alpha level by the number of possible two-by-two comparisons between groups (i.e., 0.050/3 = 0.017).

Univariate and multivariate linear regression models were estimated to identify significant predictors for glycemic outcomes (i.e., HbA1c, %TIR, %TBR < 70 mg/dL, %TAR > 180 mg/dL, and CV). In particular, we tested the influence of the following covariates: gender, age, BMI Z-score, duration of disease, duration of use of the current CSII system, use of HCL systems, and daily sensor use. The results were expressed as regression coefficient (B), 95% confidence interval, and *p*-value. Statistical analyses were performed using IBM SPSS for Windows, Version 22 (Armonk, NY, USA, IBM Corp.). A *p*-value <0.05 was considered to be statistically significant.

## 3. Results

**Figure 1:**
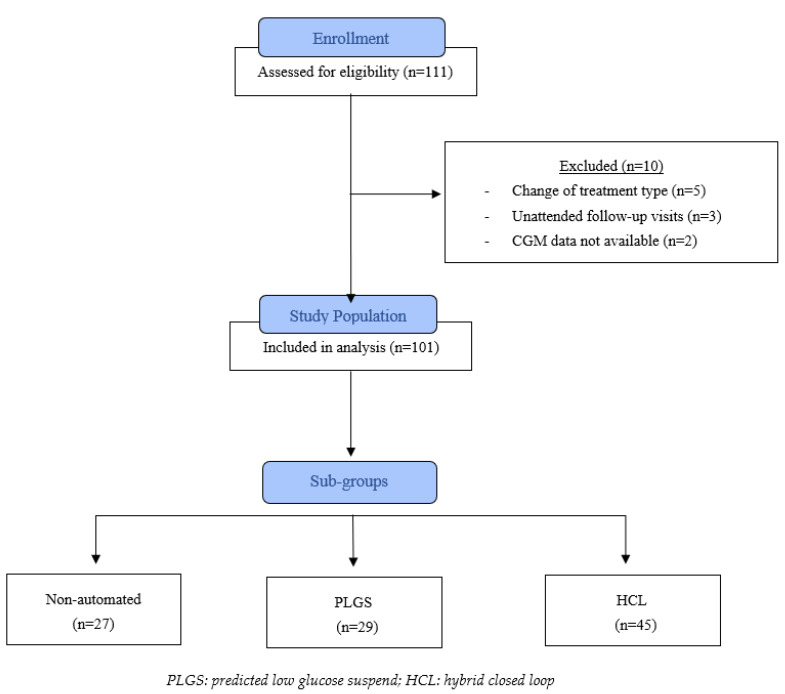
Flow diagram for patient recruitment.

Of the 111 study participants initially enrolled according to inclusion and exclusion criteria, 10 dropped-out during the 12-month study period (3 patients did not attend quarterly follow-up appointments, 5 patients changed type of treatment, and glucose data from 2 patients were unavailable). A summary of patient selection and exclusions is shown in Figure 1.

Our study population consisted of a cohort of 101 patients with a mild prevalence of male subjects (54.5%). At the time of enrollment, mean age of study participants was 12.5 ± 3.5 years, duration of disease was 5.6 ± 3.1 years, and mean BMI Z-score was 0.49 ± 0.91. Twenty-seven patients (26.7%) had a non-automated insulin pump, 29 (28.7%) used PLGS, and 45 (44.6%) were on an HCL system. Average daily insulin dose was 0.84 ± 0.20 IU/kg, distributed into 57.6 ± 11.7% basal delivery and 42.4 ± 11.7% bolus. Acute complications (e.g., DKA episodes and severe hypoglycemic events) were not reported in all patient subgroups, as well as other side effects during the entire study period.

### 3.1. Comparison of Devices

Anthropometric, clinical, and glycemic data on three subgroups of patients using different CSII systems aggregated for the entire study period are summarized in Table 1. The ANOVA test revealed significant differences in the following variables between the three subgroups: duration of use of the current device (*p* < 0.001), HbA1c (*p* = 0.040), %TBR 54–70 mg/dL (*p* = 0.035), %TBR < 70 mg/dL (*p* = 0.031), %TIR (*p* = 0.001), %TAR > 180 mg/dL (*p* = 0.007), %TAR > 250 mg/dL (*p* = 0.002), GMI (*p* = 0.028), mean blood glucose (*p* = 0.028), SD blood glucose (*p* < 0.001), CV (*p* = 0.001), basal percentage (*p* < 0.001), and bolus percentage (*p* < 0.001). A borderline significant difference of %TBR < 54 mg/dL levels (*p* = 0.057) between the three subgroups was also found.

**Figure 2:**
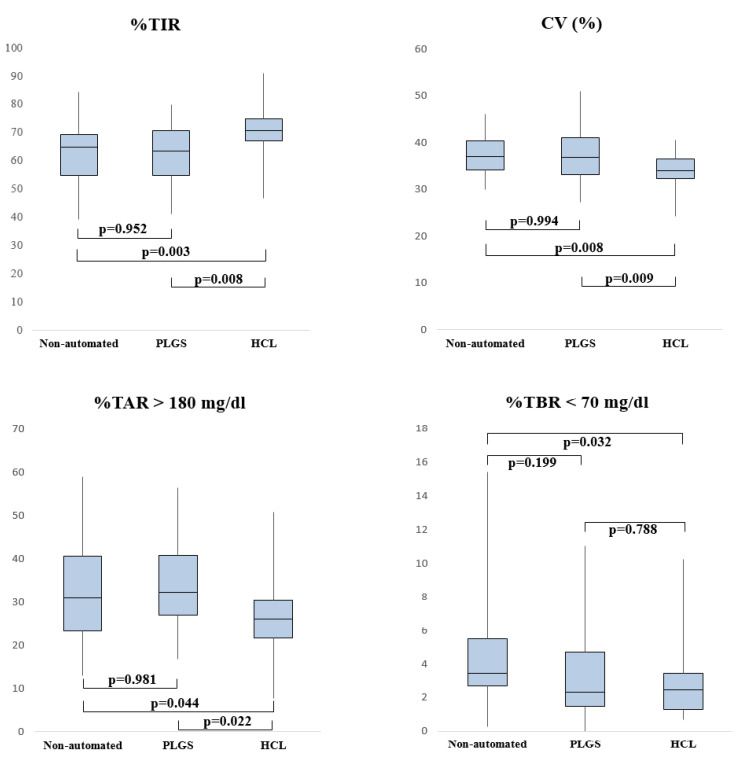
Boxplots illustrating the distribution of the main CGM metrics (%TIR, %TAR > 180 mg/dL, %TBR < 70 mg/dL, CV) in two-by-two comparisons between three different subgroups.

Two-by-two comparisons between subgroups are described in Figure 2. When compared with the non-automated group, patients using HCL systems showed higher levels of %TIR (*p* = 0.003), lower CV (*p* = 0.008), and nearly significantly lower %TBR < 70 mg/dL (*p* = 0.032) and %TAR > 250 mg/dL (*p* = 0.021). Similarly, comparing HCL to PLGS patients, we found higher %TIR (*p* = 0.008), lower CV (*p* = 0.009), and lower %TAR > 250 mg/dL (*p* = 0.007), while %TAR > 180 mg/dL (*p* = 0.022) tended to lower levels. No differences between PLGS and the non-automated group were seen. Finally, HCL systems showed a lower basal amount compared to PLGS (*p* < 0.001) and non-automated (*p* < 0.001) devices.

### 3.2. Influence of Covariates on Glycemic Outcomes

Univariate linear regression models showed that patients with higher daily sensor use achieved better glycemic outcomes in term of %TIR (*p* = 0.004), %TAR > 180 mg/dL, and HbA1c levels (*p* = 0.056). Use of HCL systems was closely associated to higher %TIR (*p* < 0.001) and lower levels of CV (*p* < 0.001), %TBR < 70 mg/dL (*p* = 0.052), and %TAR > 180 mg/dL (*p* = 0.002). These findings were strengthened by multiple linear regression models, which confirmed the association of daily percentage of sensor use with %TIR (*p* = 0.031) and %TAR >180 mg/dL (*p* = 0.015), and of the use of HCL systems with %TIR (*p* < 0.001) (Table 2), %TAR > 180 mg/dL (*p* = 0.002), %TBR < 70 mg/dL (*p*=0.029), and CV (*p* < 0.001) (Table 3). None of the remaining covariates including gender, age, BMI Z-score, duration of disease, or duration of use of the current CSII system showed any influence on glycemic outcomes.

## 4. Discussion

Our real-world evidence study compared effectiveness, expressed as the achievement of specific glycemic outcomes, among different technologies applied to CSII therapy, which are currently used in pediatric clinical practice. Our results revealed a clear superiority of HCL systems versus other technologies as demonstrated by higher levels of time spent in the target glucose range and reduction of both hypoglycemic and hyperglycemic events over a 1-year period. Randomized clinical trials conducted on pediatric patients in both controlled settings, such as hospitals or diabetes camps, and free-living conditions already revealed the efficacy of HCL systems [18,19,20]. All these studies considered the variation of HbA1c levels as one of the main outcomes to define improvement in glycemic control. Our findings are consistent with a recent systematic review and meta-analysis evaluating age-mixed studies that reported that the use of HCL allowed the achievement of increased TIR levels and reduced time in hypo- and hyperglycemia in comparison with non-automated insulin pumps and an SAP system with LGS function [21]. Another systematic review and meta-analysis investigating studies only in pediatric patients also confirmed the primacy of HCL systems in terms of time spent in the target glycemic range and reduction of hyperglycemia and hypoglycemia [22].

The introduction of SAP-supporting PLGS function already brought significant benefits in the management of T1D [23]. Our study, by revealing no differences in time spent in glucose values < 70 mg/dL if compared with HCL, confirmed the effectiveness of PLGS systems in terms of prevention of hypoglycemic events. However, time spent in hyperglycemia and short-term glycemic variability, which are well known factors associated to long-term complications of diabetes, are not adequately addressed by these algorithms. The most recent HCL systems filled this gap, by introducing the automatic modulation of the basal insulinization rate and delivery of correction boluses in the case of hyperglycemia, along with other features including specific settings for bedtime and physical activity [24]. In particular, HCL systems have been demonstrated to be related to optimum glycemic stability during night-time when the role of exogenous factors influencing glucose homeostasis are minimized [18,19].

Our data showed that HCL use allows to reduce the short-term glycemic variability assessed by glucose sensor CV. This finding is consistent with data from other randomized controlled trials and observational studies [25,26,27,28,29]. The role of glycemic variability is currently well recognized as an independent risk factor in the pathogenesis of long-term complications of diabetes [30]. Recommendations on clinical targets for continuous glucose monitoring data interpretation suggest that CV is the most suitable glucose metric to identify short-term glycemic variability and levels should be maintained below 36% [31]. Some studies recommend that lower CV targets (<33%) enhance protection against hypoglycemia for patients on insulin therapy [32].

A regular use of CGM sensors, evidenced by high daily percentages, was identified as another predictor of good glycemic control in our study population. Continuous monitoring of glucose is well known to positively influence the management of diabetes and the achievement of optimal glycemic outcomes in children and adolescents with T1D [33,34]. This aspect has been further marked with the advent of technology, allowing the interaction between CGM systems and insulin pumps. In young patients using HCL devices, increasing sensor wear time and time spent in Auto Mode have been found to be associated with better HbA1c and TIR levels [35]. However, some barriers that hinder the regular use of glycemic sensors still exist. The frequent need of calibrations for some devices, issues related to durability and adhesion, and skin reactions due to prolonged exposure to chemical and mechanical agents contained in the adhesives, are the main causes of sensor use discontinuation [36,37,38,39].

Given the superiority in glycemic outcomes, as demonstrated by our study, HCL systems have recently become the first choice for pediatric patients with T1D approaching the use of technology. However, HCLs currently available in most countries are characterized by the presence of a potentially inconvenient external system. As known, the discomfort of wearing diabetes devices is one of the most common causes of CSII treatment discontinuation, especially during adolescence. Therefore, although patch pumps are not currently connected to glycemic sensors, their use should be seriously considered for those patients who feel disturbed by how devices look on their bodies. Finally, PLGS systems are generally recommended for young children with low daily insulin dose and a high risk of hypoglycemic events, who do not meet the age criteria for HCL devices.

The main strengths of our study are its longitudinal design, the real-world setting, and the duration of the study period. The inclusion criteria of at least 6 months of use of the current device at the time of the enrollment allowed to exclude any bias related to poor experience with the use of the insulin pump.

The presence of devices containing different algorithms within the same subgroup represents a limitation of the present study, particularly in the evaluation of total daily insulin dose and its distribution between basal and bolus rates. Dietary interventions in different subgroup of CSII users are not included in the study design. Furthermore, other relevant aspects such as psychological and economic impact related to different CSII systems were not considered in our study. Bisio et al. demonstrated that psychological and behavioral issues related to pediatric diabetes including anxiety, fear of hypoglycemia, and sleep disturbances, which represent a great burden of the disease, benefited greatly from technological progress [40]. Finally, despite the high costs for public health systems, the newest therapeutic devices have been shown to be cost-effective in comparison to traditional treatments over patient lifetimes, as a result of a reduction of morbidity mainly attributable to long-term complications [41,42].

## 5. Conclusions

The development of technological devices resembling ever more the concept of an artificial pancreas has been a game changer in the management of T1D in pediatric age. Our real-world data confirm the superiority of HCL systems in terms of improvement of time spent in the target glucose range, prevention of hypoglycemia, and reduction of glycemic variability. However, accurate knowledge of the device on the part of patients and caregivers, which also involves regular use of CGM systems, is crucial to achieve the desired therapeutic goals. Therefore, educational programs addressed to the proper use of technological devices has a primary role for the achievement of desirable therapeutic goals.

## Figures and Tables

**Table 1:** Comparison of anthropometric and clinical data among patients with different CSII systems. Indicators of glycemic control are expressed as the mean and standard deviation of the aggregated data of each quarterly follow-up visit.

	Non Automated	PLGS	HCL	*p*-Value
**Gender**				0.621
Male	14 (51.8%)	17 (58.6%)	23 (51.1%)	
Female	13 (48.1%)	12 (41.3%)	22 (48.8%)	
**Age (years)**	13.2 ± 3.3	12.6 ± 3.8	12.1 ± 3.5	0.416
**Duration of diabetes (years)**	6.3 ± 2.9	5.2 ± 2.9	5.5 ± 3.3	0.389
**BMI z-score**	0.48 ± 0.81	0.26 ± 0.78	0.64 ± 1.01	0.226
**Duration of use CSII (years)**	2.8 ± 1.7	2.8 ± 1.9	1.4 ± 1.2	<0.001 *
**HbA1c (%)**	6.7 ± 0.5	7.1 ± 0.8	7.1 ± 0.6	0.040 *
**%TBR < 54 mg/dL**	1.1 ± 1.4	0.6 ± 1	0.5 ± 0.9	0.057
**%TBR 54–70 mg/dL**	3.6 ± 2.5	2.8 ± 1.9	2.4 ± 1.4	0.035 *
**%TIR 70–180 mg/dL**	61.7 ± 11.6	62.6 ± 10.4	70.2 ± 8.7	0.001 *
**%TAR 180–250 mg/dL**	24.5 ± 7.1	24.6 ± 5.9	21.8 ± 6.5	0.099
**%TAR > 250 mg/dL**	8. 9 ± 6.9	9.4 ± 6	5.3 ± 3.8	0.002 *
**GMI (%)**	7.1 ± 0.4	7.1 ± 0.4	6.9 ± 0.3	0.028 *
**Use of sensor (%)**	80.9 ± 22.6	74.5 ± 19.6	82.4 ± 15.1	0.204
**Mean glucose levels (mg/dL)**	158.2 ± 20.6	160.5 ± 16.1	150.8 ± 12.6	0.028 *
**SD glucose levels (mg/dL)**	59.7 ± 10.7	60.1 ± 10.9	51.7 ± 7.6	<0.001 *
**CV (%)**	37.5 ± 4.4	37.3 ± 5.4	34 ± 3.9	0.001 *
**Daily insulin dose (IU/kg)**	0.82 ± 0.16	0.81 ± 0.18	0.9 ± 0.2	0.422
**Basal insulin (%)**	63.8 ± 6.8	62.9 ± 11.2	50.4 ± 10.4	<0.001 *
**Bolus (%)**	36.1 ± 6.6	37.1 ± 11.2	49.6 ± 10.4	<0.001 *

BMI: body mass index; CSII: continuous subcutaneous insulin infusion; CV: coefficient of variation; HbA1c: glycated hemoglobin; GMI: glucose management indicator; SD: standard deviation; %TAR 180–250 mg/dL: time above range between 180 and 250 mg/dL; %TAR > 250 mg/dL: time above range > 250 mg/dL; %TBR < 54 mg/dL: time below range < 54 mg/dL; %TBR 54–70 mg/dL: time below range between 54 and 70 mg/dL; %TIR 70–180 mg/dL: time in range between 70 and 180 mg/dL. * significant *p*-values.

**Table 2:** Results of multivariate logistic regression models for %TIR.

	Adjusted OR	95% C.I.	*p* Value
Age (years)	−0.91	−0.69–0.51	0.765
Gender	2.53	−1.38–6.44	0.203
BMI z-score	−2.09	−4.30–0.11	0.063
Duration of diabetes	−0.38	−1.11–0.34	0.296
Duration of CSII use	0.69	−0.66–2.04	0.313
Sensor daily use (%)	0.12	0.01–0.23	0.031 *
HCL use	8.58	4.27–12.90	<0.001 *

BMI: Body Mass Index; HCL: hybrid closed loop. * significant *p*-values.

**Table 3:** Results of multivariate logistic regression models for CV.

	Adjusted OR	95% C.I.	*p* Value
Age (years)	−0.28	−0.60–0.02	0.073
Gender	0.25	−1.59–2.10	0.784
BMI z-score	−0.66	−1.77–0.43	0.231
Duration of diabetes	+0.36	−0.18–0.74	0.061
Duration of CSII use	−0.35	−0.93–0.21	0.213
Sensor daily use (%)	−0.04	−0.08–0.008	0.098
HCL use	−3.45	−5.58–−1.32	<0.002 *

BMI: body mass index; HCL: hybrid closed loop. * significant *p*-values.

## Data Availability

The data that support the findings of this study are not publicly available due to privacy reasons of research participants, but are available from the corresponding author upon reasonable request.
